# Artificial urinary sphincter for urinary incontinence after radical prostatectomy: a historical cohort from 2004 to 2015

**DOI:** 10.1590/S1677-5538.IBJU.2016.0244

**Published:** 2017

**Authors:** Augusto Cesar Soares dos Santos, Luíza de Oliveira Rodrigues, Daniela Castelo Azevedo, Lélia Maria de Almeida Carvalho, Mariana Ribeiro Fernandes, Sandra de Oliveira Sapori Avelar, Maria da Glória Cruvinel Horta, Silvana Márcia Bruschi Kelles

**Affiliations:** 1 Grupo de Avaliação de Tecnologia em Saúde, Unimed BH, MG, Brasil;; 2 Núcleo de Avaliação de Tecnologia em Saúde, Hospital das Clínicas, Universidade Federal de Minas Gerais (UFMG), MG, Brasil

**Keywords:** Urinary Incontinence, Prostatectomy, Prostatic Neoplasms, Urinary Sphincter, Artificial

## Abstract

This study aimed to retrospectively evaluate a cohort of patients with prostate cancer and persistent urinary incontinence after radical prostatectomy. From January 2004 to December 2015, eighty-six individuals were identified to have received an AUS implant, provided by a private nonprofit HMO operating in Belo Horizonte, Brazil. On total, there were 91 AUS implants, with a median interval between radical prostatectomy and AUS implant of 3.6 years (IQR 1.9 to 5.5). The rate of AUS cumulative survival, after a median follow-up of 4.1 years (IQR 1.7-7.2 years), was 44% (n=40). The median survival of AUS implants was 2.9 years (IQR 0.5-7.9 years). Thirty-seven AUS implants (40.7%) resulted in grade III surgical complications. There were 5 deaths at 2.1, 4.7, 5.7, 5.7 and 6.5 years of follow-up, but none due to causes directly associated to the AUS implant. Persistent severe incontinence was documented in 14 (15.3%) additional patients. From the 51 AUS implants which resulted in grade III surgical complications or persistent severe incontinence, 24 (47.1%) underwent surgical revisions. Explantation of the sphincter or its components was observed in 6 cases (25.0%). Mechanical failure, described as fluid loss and/or inability to recycle the AUS device, was observed in 4 devices (16.7%). In conclusion, although AUS implants are recommended as the gold-standard treatment of severe urinary incontinence after prostatectomy, the observed high rates of malfunction and grade III adverse events are a matter of concern warranting further assessment on the safety and efficacy of these devices.

## INTRODUCTION

Currently, prostate cancer is the leading type of cancer in men worldwide, with a global estimated incidence of 1.4 million cases a year (1). In spite of the risks of urinary incontinence, and other adverse events such as impotence, radical prostatectomy is still the most frequently performed treatment for this condition (2).

Urinary incontinence, the involuntary urethral loss of urine, can be caused by radical prostatectomy through a direct injury of the urethral sphincter or as a consequence of bladder denervation, resulting in bladder dysfunction such as detrusor overactivity (3). While a small amount of incontinence may not cause problems, larger degrees of incontinence can lead to major impact on a patient’s quality of life (4). In these cases, when incontinence persists despite conservative therapy, the implantation of an artificial urinary sphincter (AUS) may be recommended (5, 6).

An AUS consists of three silicone components: a cuff, a balloon reservoir, and a pump. Each of these components is attached to a length of silicone tubing and connected together during the surgical implant procedure (7). In spite of its known efficacy in the management of persistent urinary incontinence, studies have reported disastrous complications resulting in early device removal and an increased rate of surgical revisions (8-18). Therefore, this study aimed to retrospectively evaluate a series of cases of AUS implants in patients with persistent urinary incontinence after radical prostatectomy, at a private nonprofit health maintenance organization (HMO) in Brazil.

## PATIENTS AND METHODS

This study consisted of a convenience sample of individuals with persistent urinary incontinence after radical prostatectomy performed to treat prostate cancer. We retrospectively collected data from individuals who had an AMS800® AUS device implanted from January 2004 to December 2015, while they were being provided healthcare assistance by a private nonprofit HMO operating in Belo Horizonte, Brazil. Data was collected from AUS implants performed in 15 different hospitals in the Belo Horizonte metropolitan region, the third largest metropolitan area of Brazil. Patients were excluded if they had a history of any urological surgical procedure other than radical prostatectomy.

The primary outcomes of this study were the assessment of grade III surgical complications following AUS implantation, which were defined, according to the Clavien-Dindo classification score, as any deviation from the ideal postoperative course that is not inherent in the procedure and does not comprise a failure to cure requiring surgical, endoscopic or radiological intervention (19, 20). The need for surgical revision was defined as the first repeat operation on the AUS, including due to total or partial explantation or to mechanical failure. Demographic information collected for each patient included age, date of the radical prostatectomy, history of previous radiotherapy, date of AUS implantation, costs, need for revision or removal of the device. Data was extracted from an administrative database, using the software Oracle Business Intelligence®.

After a descriptive analysis of the data, patients were divided in two groups, according to their history of radiotherapy. Continuous data were expressed as medians and interquartile range (IQR) or means and standard deviation (SD), when appropriate. Dichotomous variables were compared using two-sided Fisher’s exact test. The level of significance was set at p<0.05. Kaplan-Meier estimates of survival curves were built using the software STATA 13.1 (Stata Corp, College Station, TX, USA).

This historical cohort resulted in no interventions, neither during the course of the instituted treatment nor after the observed outcome. Privacy of subjects and the confidentiality of their personal information were handled in accordance to the ethical principles of the Declaration of Helsinki. This study was approved by the local ethics committee.

## RESULTS

**Figure 1:**
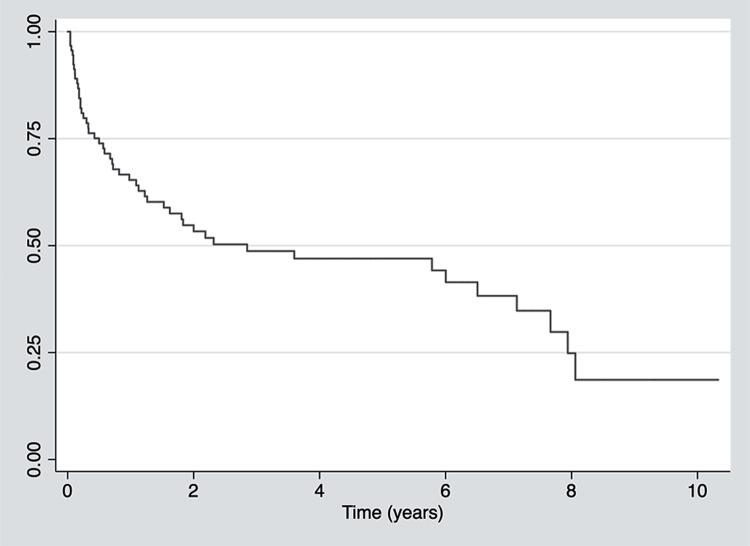
Artificial urinary sphincter survival curve.

From January 2004 to December 2015, 86 men were identified to have received an AUS implant after radical prostatectomy. The mean age at the time of the AUS implantation was 69.5 years (range 47.5 to 86.0 years). Five patients (5.8%) underwent a second AUS implant, due to AUS malfunction, resulting in a total of 91 devices. Total device costs were estimated in roughly US$1.000.000.00 or US$11.628.00 per patient. Implants were performed in 15 different hospitals by 28 different surgeons. The median interval between radical prostatectomy and AUS implant was 3.6 years (IQR 1.9-5.5 years). The rate of AUS cumulative survival, after a median follow-up of 4.1 years (IQR 1.7-7.2 years), was 44% (n=40). The median survival of AUS implants was 2.9 years (IQR 0.5-7.9 years), as shown in Figure-1.

Thrity-seven AUS implants (40.7%) resulted in grade III surgical complications, distributed as follows: scrotal abscess (n=10, 11.0%); sepsis due to prosthesis infection (n=9; 9.9%); urethral fistula (n=7; 7.7%); urethral erosion (n=6; 6.6%); urethral stenosis (n=3; 3.3%); acute postoperative urinary retention (n=1; 1.1%); testicular torsion (n=1; 1.1%). Persistent severe incontinence was documented in 14 (15.3%) additional patients. There were 5 deaths at 2.1, 4.7, 5.7, 5.7 and 6.5 years of follow-up, but none due to causes directly associated to the AUS implants.

From the 51 AUS implants which resulted in grade III surgical complications or persistent severe incontinence, 24 (47.1%) demanded surgical revisions. The median time to first revision was 8.1 months (IQR 2.2-21.9 months). The revisions were triggered by: failure of the cuff (n=8; 33.3%); the need to replace the balloon reservoir (n=3; 12.5%); the need to install a second cuff (n=2; 8.3%) or to reposition of pumps (n=1; 4.2%). Explantation of the sphincter or its components was observed in 6 cases (25.0%). Mechanical failure, described as fluid loss and/or inability to recycle the AUS device, was observed in 4 devices (16.7%).

Twelve (14.0%) patients were exposed to radiotherapy (RT) before the implant of an AUS. There were no significant statistical differences for the rate of surgical complications (p=0.7) and the need for surgical revisions (p=0.6) after patient stratification according to their history of prior RT (Table-1).

**Table-1:** Frequency of surgical revisions and grade III surgical complications after AUS implantation according to the history of previous radiotherapy

	Previous radiotherapy (n=12)	No previous radiotherapy (n=74)	P*
Presence of grade III surgical complications - n(%)	4 (33%)	33 (44%)	0.7
Underwent surgical revision - n(%)	2 (16%)	22 (30%)	0.6

*Two-sided Fisher's exact test.

## DISCUSSION

Therapeutic strategies for urinary incontinence after prostatectomy include conservative treatment and pharmacotherapy (21, 22). For those who have persistent severe urinary incontinence in spite of these measures, surgical options, such as the use of transurethral bulking agents, perineal slings or AUS implants, are usually recommended. Currently, AUS implants are considered the gold standard surgical option (23, 24). Nevertheless, studies evaluating AUS efficacy and long term complications are scarce, especially in low and middle-income countries, such as Brazil.

In this context, this study retrospectively evaluated a cohort of patients with persistent urinary incontinence after radical prostatectomy that underwent AUS implantation. In our cohort, the rate of AUS cumulative survival, after a median follow-up of 4.1 years, was 44.0% (n=40). Thirty-seven AUS implants resulted in grade III surgical complications, while fourteen resulted in persistent severe incontinence. Our median time to first revision was 8.1 months (IQR 2.2-21.9 months) and the rate of surgical revision was 26.4% (n=24). When compared to our cohort, Ravier et al. (18), reported a longer median time to first revision (11.7 months), with a similar rate of surgical revisions (31.0%). In that cohort, with 122 patients, there were no revisions due to mechanical failure of the device, differently from what was observed in 4 of our patients.

Ravier et al. (18) reported an overall rate of continence of 68.9%. Other studies reported a long-term complete continence of only 20.0% (14) and surgical revision rates of 22.0% (10) and 25.0% (25). In similarity to our results, in 2012 Wang et al. (15) reported, after a median follow-up of 52 months (4.3 years), the need for at least one intervention in 53.0% of his sample. Revisions occurred after a median time of 20.1 months and were most commonly motivated by recurrent incontinence (56.7%), mechanical malfunction (22.0%) and infection or erosion (18.6%).

In the face of the high rates of AUS complications reported in the medical literature, some authors have tried to identify possible risk factors. In 2015, Hird et al. (16) published a study suggesting that despite the recent improvements in radiation treatment techniques and equipment, previous exposure to radiotherapy could still be considered a risk factor for surgical complications after AUS implants. In our study, complications were numerically more prevalent in patients without previous history of radiotherapy, however this observed difference was not statistically significant. Similar results were reported by Kim et al. (26), which also didn´t find significant differences in AUS complication rates according to previous RT exposure. In spite of these results, our findings should be analysed in the context of lack of power to address the impact of RT in this population since only 12 (14.0%) patients in our cohort were previously exposed to RT. Other limitations of the present study is its retrospective, non-randomized and uncontrolled design. Because of its nonconcurrent nature and its data source limited to an administrative database, we did not have access to clinical data, such as time between RT and AUS implantation, radiation dose or RT type. We also didn’t have access to quality-of-life or functional parameters related to clinical outcomes of the AUS. Finally, in this study, 91 AUS implants were performed in 15 hospitals by 28 different surgeons, raising the question whether suboptimal surgical expertise might have influenced our results. In spite of these limitations, as far as we know, this is the first study to assess AUS implants in Brazil.

In conclusion, although AUS implants are recommended as the gold-standard treatment for severe persistent urinary incontinence after prostatectomy, the observed high rates of device malfunction and grade III surgical complications are a matter of concern warranting further assessment on the safety and efficacy of these devices.

## ARTICLE INFO

Int Braz J Urol. 2017; 43: 150-4
